# Self-Reported Sleep Disturbances in Patients with Dissociative Identity Disorder and Post-Traumatic Stress Disorder and How They Relate to Cognitive Failures and Fantasy Proneness

**DOI:** 10.3389/fpsyt.2014.00019

**Published:** 2014-02-19

**Authors:** Dalena van Heugten – van der Kloet, Rafaele Huntjens, Timo Giesbrecht, Harald Merckelbach

**Affiliations:** ^1^Faculty of Psychology and Neuroscience, Maastricht University, Maastricht, Netherlands; ^2^Department of Clinical Psychology, University of Groningen, Groningen, Netherlands

**Keywords:** dissociative symptoms, sleep quality, unusual sleep experiences, cognitive failures, fantasy proneness

## Abstract

Sleep disturbances, fantasy proneness, cognitive failures, and dissociative symptoms are related to each other. However, the co-occurrence of these phenomena has been primarily studied in non-clinical samples. We investigated the correlations between these phenomena in dissociative identity disorder (DID) patients, post-traumatic stress disorder (PTSD) patients, and healthy controls. Both patient groups reported more sleep problems and lower sleep quality and displayed higher levels of fantasy proneness and cognitive failures than controls. However, the two patient groups did not differ with regard to these variables. Moreover, a higher level of unusual sleep experiences tended to predict participants belonging to the DID group, while specifically a lower sleep quality and more cognitive failures tended to predict participants belonging to the PTSD group.

## Introduction

In psychopathology, dissociation typically refers to a disturbance in the normal integration of thoughts, feelings, and experiences into consciousness and memory. As dissociative symptoms are prevalent in both normal and clinical populations, dissociation has commonly been conceptualized as ranging on a continuum, from non-pathological manifestations of daydreaming to more severe disturbances typical of dissociative disorders (1), which encompass dissociative amnesia, dissociative fugue, depersonalization disorder (DPD), and dissociative identity disorder (DID) (2).

Epidemiological studies among psychiatric inpatients and outpatients have yielded prevalence rates of dissociative disorders exceeding 10% (3–6), and a recent study among women in the general population reported a prevalence rate of 18.3% for lifetime diagnoses of a dissociative disorder (7). However, dissociative symptoms are not limited to the dissociative disorders. Certain diagnostic groups, notably patients with borderline personality disorder, post-traumatic stress disorder (PTSD), obsessive–compulsive disorder (8), and schizophrenia-spectrum disorder (9) also display heightened levels of dissociation (10, 11).

A widely held notion about the etiology of dissociative symptoms is that they serve a defensive function in that they help to cope with traumatic memories. Thus, many clinicians assume that dissociative symptoms are intimately linked to a history of traumatic childhood events (12). However, this conceptualization remains silent as to how precisely trauma might contribute to dissociative symptoms.

Recent studies have pointed to the role of sleep disturbances in dissociative symptoms (13). This research line is not incompatible with the traumatogenic approach to dissociative symptoms. Thus, traumatic experiences may disrupt sleep, which in turn may contribute to or exacerbate dissociative symptoms. In our recent review, we (14) concluded that data from 23 studies of clinical and non-clinical samples provide strong support for a link between dissociative experiences and sleep problems. This link is evident across a range of sleep-related phenomena, including waking dreams, nightmares, and hypnagogic (occurring while falling asleep) and hypnopompic (occurring while awakening) hallucinations. In support of this hypothesis, studies of the association between dissociative experiences and sleep disturbances have generally yielded modest correlations (in the range of 0.30–0.55), implying that unusual sleep experiences and dissociation are moderately related constructs [see also Ref. (15)].

Apart from their links with traumatic experiences and sleep disturbances, dissociative symptoms are related to cognitive failures and fantasy proneness (16). Individuals suffering from dissociative symptoms typically score high on measures of fantasy proneness, which is a disposition to engage in extensive and vivid fantasizing (10). Furthermore, individuals scoring high on dissociation report more cognitive failures (i.e., everyday slips and lapses) compared with individuals scoring low on dissociation. People who frequently make such slips and lapses often mistrust their own cognitive capacities and tend to overvalue the hints and cues provided by others, making them susceptible for suggestive information or manipulation (16, 17).

One interpretation of these associations is that individuals with sleep problems experience intrusions of sleep phenomena (i.e., dreamlike experiences) into waking consciousness, which may foster dissociative symptoms. These sleep problems may have originated from distressing trauma-related memories, a genetic propensity (18), or other unknown influences. Furthermore, sleep disturbances may undermine cognitive efficiency by degrading memory and attentional control and fuel imaginative mentation, both of which are important constituents of dissociative symptoms.

Dissociative symptoms have the reputation of being refractory to treatment interventions (19). The recent research focus on sleep disturbances may contribute to the development of effective sleep hygiene interventions for patients with dissociative symptoms. However, before exploring this research avenue, it is important to ascertain in clinical groups with known dissociative symptomatology that sleep disturbances occur and are linked to dissociative symptoms and its correlates. Therefore, we investigated how cognitive failures, fantasy proneness, and sleep are related to dissociative symptoms in a sample of DID patients, patients with PTSD, and healthy controls.

## Materials and Methods

Twelve female DID patients (mean age: 42 years, SD = 11.8), 27 female PTSD patients who reported childhood sexual and/or physical abuse (mean age: 42 years, SD = 13.1), and 55 healthy female controls (mean age: 42 years, SD = 13.1) took part in the study. The choice for female patient groups was made because of a practical reason: DID is mostly prevalent in women. The DID group had a mean of 5.3 years of post-elementary education (SD = 1.47), the PTSD group had studied for a mean of 4.15 years (SD = 1.25), and the controls had finished a mean of 5.9 years of education (SD = 1.18). Both DID and PTSD patients were recruited from treatment settings in The Netherlands and Belgium by asking clinicians to invite patients to participate. The clinician’s diagnosis of DID was verified with the Structured Clinical Interview for DSM-IV Dissociative Disorders [SCID-D; (20)] by the second author. Mean length of treatment for DID was 8.61 years (SD = 5.6). DID was always the main reason for patients to be in treatment; all had a history of multiple hospitalizations and a relatively chronic course. Mean number of reported identities was 28 (range: 4–39, with one outlier of 196). PTSD status was verified with the Dutch version of the clinician-administered PTSD Scale (21) by the second author. Mean length of treatment for PTSD was 2.58 years (SD = 3.2). PTSD was always a primary diagnosis.

Most participants used medication during the time of the study. In the DID group, medications were anxiolytics (i.e., benzodiazepines; *N* = 6), antidepressants (*N* = 8), antipsychotics (*N* = 1), and pain medication (*N* = 3). In the PTSD group, medications were anxiolytics (i.e., benzodiazepines; *N* = 13), antidepressants (*N* = 14), pain medication (*N* = 1), and beta-adrenergic blockers (*N* = 1).

Participants in the control group were matched on gender and age^1^. They were community volunteers.

All participants received oral information prior to enrollment in the study, after which they gave written informed consent. Participants completed a test battery including the Dissociative Experiences Scale [DES; (1)], which has three subscales measuring absorption, depersonalization/derealization, and amnesia; the Iowa Sleep Experiences Survey [ISES; (15)], which has two subscales measuring general sleep experiences and lucid dreaming, the Pittsburgh Sleep Quality Inventory [PSQI; (22)], the Creative Experiences Questionnaire, which is an index of fantasy proneness [CEQ; (23)], and the Cognitive Failures Questionnaire [CFQ; (24)], which is a measure of cognitive efficiency. The current data were collected as part of a larger study (25).

## Results

The groups differed in amount of years of education. Using ANOVA, we found that this difference was significant between all three groups [*F*(2, 94) = 17.31, *p* < 0.001] with the PTSD group scoring lower than the DID and control groups. Table 1 summarizes the psychometric test data of the three groups. The groups differed significantly from each other with regard to the total and subscale dissociation (DES) scores, with the exception of the absorption subscale on which the DID group did not score higher than the PTSD group. For all other measures, a significant difference was found between DID and controls and between PTSD and controls, but not between the two patient groups.

**Table 1:** **Mean scores (standard deviations) on dissociation (DES), unusual sleep experiences (ISES), sleep quality (PSQI), fantasy proneness (CEQ), and cognitive failures (CFQ) of the DID (*N* = 12), PTSD (*N* = 27), and control (*N* = 55) groups**.

	DID	PTSD	Control	*F*	*p*	*C*’s α
**DES**
Total^a^^b^^c^	44.99 (13.99)	26.23 (16.55)	7.01 (5.23)	72.73	<0.001	0.97
Absorption^a^^c^	43.88 (17.55)	35.64 (20.51)	11.54 (8.97)	40.85	<0.001	0.91
Amnesia^a^^b^^c^	37.81 (13.97)	14.03 (14.75)	2.34 (2.90)	71.28	<0.001	0.92
Depersonalization^a^^b^^c^	49.44 (18.05)	22.04 (22.14)	2.70 (4.60)	62.08	<0.001	0.93
**ISES**
Total^a^^c^	69.42 (18.69)	64.85 (21.64)	40.05 (15.07)	25.49	<0.001	0.93
General sleep^a^^c^	62.50 (17.71)	57.93 (19.96)	34.11 (12.48)	30.10	<0.001	0.93
Lucid dreaming	6.92 (3.03)	6.93 (3.73)	5.95 (3.72)	0.82	0.44	0.75
**PSQI^a^^c^**	13.92 (3.58)	14.74 (3.04)	8.93 (2.47)	45.45	<0.001	0.80
**CEQ^a^^c^**	10.17 (3.66)	8.82 (4.50)	5.64 (3.99)	9.20	<0.001	0.84
**CFQ^a^^c^**	49.75 (16.60)	53.89 (10.99)	41.35 (7.80)	14.63	<0.001	0.93

Pairwise comparisons significant at the 0.05 level (two-tailed, Bonferroni corrected) are designated as:

^a^ DID differs from control;

^b^ DID differs from PTSD;

*^c^ PTSD differs from control. *C*’s α, Cronbach’s alpha of this sample*.

Table 2 displays the Pearson product moment partial correlations between the measures. Effect sizes were medium to large. We performed backwards binary logistic regression analyses^2^ on the DID group and the PTSD group to determine which variable most likely predicted the chance of belonging to one of the psychopathology groups. We entered the DID or PTSD group as dependent variable, respectively. Then, based on our earlier ANOVA, we entered CEQ, CFQ, PSQI, and ISES as predictors in each analysis. We left out the DES, as it would bias our analyses, because dissociation forms a diagnostic determinant in DID. A summary of the regression analyses are displayed in Table 3. We found that a high score on the ISES best predicted the chance of belonging to the DID group, while higher scores on the CFQ and PSQI predicted the likelihood of belonging to the PTSD group.

**Table 2:** **Pearson product moment partial correlations between baseline measures (*N* = 94), controlling for years of education**.

	DES	ISES	PSQI	CEQ
**DES**	–	–	–	–
**ISES**	0.63**	–	–	–
**PSQI**	0.49**	0.45**	–	–
**CEQ**	0.47**	0.46**	0.22*	–
**CFQ**	0.53**	0.43**	0.43**	0.42**

*DES, Dissociative Experiences Scale; ISES, Iowa Sleep Experiences Survey; PSQI, Pittsburgh Sleep Quality Index; CEQ, Creative Experiences Questionnaire; CFQ, Cognitive Failure Questionnaire*.

*p < 0.05.

**p < 0.001.

**Table 3:** **Summary of backwards binary logistic regression analyses on DID group (*N* = 12), PTSD group (*N* = 27), and combined psychopathology group (*N* = 39)**.

Model	*B* (SE)	Wald	*p*	*R*^2^	χ^2^ (df)	*p*
**DID**
1.				0.27	14.74 (4)	0.005
CEQ	0.15 (0.09)	2.93	0.09			
CFQ	−0.04 (0.03)	1.65	0.20			
PSQI	0.18 (0.10)	3.27	0.07			
ISES	0.03 (0.02)	3.31	0.07			
2.				0.25	13.16 (3)	0.004
CEQ	0.12 (0.08)	2.09	0.15			
PSQI	0.13 (0.09)	2.07	0.15			
ISES	0.03 (0.02)	2.50	0.11			
3.				0.21	11.03 (2)	0.004
CEQ	0.10 (0.08)	1.76	0.19			
ISES	0.04 (0.02)	4.70	0.03			
4.				0.17	9.26 (1)	0.002
ISES	0.04 (0.01)	8.39	<0.01			
**PTSD**
1.				0.49	39.09 (4)	<0.001
CEQ	0.04 (0.08)	0.23	0.63			
CFQ	0.04 (0.03)	1.66	0.20			
PSQI	0.33 (0.09)	12.04	<0.001			
ISES	0.02 (0.02)	1.12	0.29			
2.				0.49	38.86 (3)	<0.001
CFQ	0.05 (0.03)	2.35	0.13			
PSQI	0.32 (0.09)	11.95	<0.001			
ISES	0.02 (0.02)	1.32	0.25			
3.				0.47	37.58 (2)	<0.001
CFQ	0.06 (0.03)	4.09	0.04			
PSQI	0.34 (0.09)	14.21	<0.001			

*DID, DID group (*N* = 12); PTSD, PTSD group (*N* = 27); ISES, Iowa Sleep Experiences Survey; PSQI, Pittsburgh Sleep Quality Index; CEQ, Creative Experiences Questionnaire; CFQ, Cognitive Failure Questionnaire*.

## Discussion

This study is – to the best of our knowledge – the first to examine the relation between self-reported sleep disturbances, fantasy proneness, and cognitive failures in patients with DID. Our aim was to ascertain in clinical groups with known dissociative symptomatology that sleep disturbances occur and are linked to dissociative symptoms and its correlates. Before we discuss the conclusions that can be drawn from our findings, it is important to emphasize some limitations of the current study. Although our sample consisted of a unique group of patients with DID, combined with a group of PTSD patients and a healthy group of controls, sample sizes of the patient groups were relatively small and limited to the female gender. This may limit the generalizability of our results. Thus, future research might want to include a larger sample of both male and female patients. Furthermore, it would have been superior if we could have conducted mediation analyses that would allow for specific testing within patient groups. Unfortunately, this was not possible due to low power issues. Therefore, using logistic regression analyses, we aimed to provide some information about the separate groups. Another important limitation of our study consists of the use of medication in our sample. As indicated, most patients were using medication during the time of the study. This is not ideal, and we would have preferred a sample that would be able to refrain from medication during our evaluations. The medication used in this sample may have had its influence on our outcome measures (dissociation, sleep quality, cognitive functioning). Unfortunately, DID and PTSD patients suffer from very severe symptomatology that consequently makes it impossible to quit medication use, even for a short period of time. Most importantly, our study relied on a cross-sectional design based on self-report measures that precludes any causal interpretations. For example, cognitive failures would be measured more reliably in experimental designs, rather than self-report. Thus, a longitudinal set-up in which patients undergoing targeted treatments for their sleep dysfunctions are followed over time would provide a more optimal starting point for testing causal hypotheses.

With these limitations in mind, the main findings of our study can be summarized as follows.

The DID group only differed from the PTSD group in the number of dissociative symptoms. Both patient groups reported more unusual sleep experiences (as measured by ISES) and worse sleep quality (as measures by PSQI) than controls, and scored more pronounced on levels of fantasy proneness and cognitive failures. Moreover, a higher level of unusual sleep experiences tended to predict participants belonging to the DID group, while specifically a lower sleep quality and more cognitive failures tended to predict participants belonging to the PTSD group.

Our findings are consistent with the hypothesis that sleep problems lead to intrusions of sleep phenomena into waking consciousness, resulting in dissociative experiences (15). Because sleep disruptions exert detrimental effects on attentional control and memory, they may contribute to the attention deficits that are typically found in patients with dissociative disorders (26). However, our data also indicate that both unusual sleep experiences and sleep problems (i.e., poor sleep quality) are not unique for patients with DID. Patients with DID and PTSD seem to share these sleep-related problems and future studies focusing on the treatment implications are warranted, as the impact of sleep quality on cognitive functioning might be substantial in these patients. The idea that DID is strongly related to PTSD fits well with our findings (27).

## Conflict of Interest Statement

The authors declare that the research was conducted in the absence of any commercial or financial relationships that could be construed as a potential conflict of interest.

## Footnotes

^1^The control group included in this manuscript partly overlaps with the healthy control group and simulator group as included in Ref. (25). The simulators were not instructed to mimic DID on the measures included in the current manuscript. The simulator and control groups did not differ on fantasy proneness (*t* = 0.03, *p* = 0.98), age (*t* = 0.32, *p* = 0.75), or level of education [*t* = 0.04, *p* = 0.40, for more information see Ref. (25)].

^2^We performed analyses both with and without number of education years as possible confounder. Education did not prove to make a significant difference in outcome scores. Therefore, we decided to present only the original analyses without education.
