# Corrigendum: Self-Reported Sleep Disturbances in Patients with Dissociative Identity Disorder and Post-Traumatic Stress Disorder and How They Relate to Cognitive Failures and Fantasy Proneness

**DOI:** 10.3389/fpsyt.2014.00112

**Published:** 2014-09-01

**Authors:** Dalena van Heugten – van der Kloet, Rafaele Huntjens, Timo Giesbrecht, Harald Merckelbach

**Affiliations:** ^1^Faculty of Psychology and Neuroscience, Maastricht University, Maastricht, Netherlands; ^2^Department of Clinical Psychology, University of Groningen, Groningen, Netherlands

**Keywords:** dissociative symptoms, sleep quality, unusual sleep experiences, cognitive failures, fantasy proneness

## Corrigendum

Page 2, Material and Methods, paragraph 1

This should read

“(…) 27 female PTSD patients who reported childhood sexual and/or physical abuse (mean age: 40 years, SD = 12.9), and (…) Education was assessed on a scale from 1 (low) to 7 (high) (1). The DID group had a mean of 5.25 (SD = 1.48) years of education, the PTSD group had studied for a mean of 5.04 years (SD = 0.90), and the controls had finished a mean of 5.82 years of education (SD = 0.98).”

Page 2, Results, paragraph 1, third sentence

This should read *F*(2, 93) = 5.64, *p* < 0.001

Table 3, DID group model 4, *R*^2^

This should read *R*^2^ = 0.18

## Conflict of Interest Statement

The authors declare that the research was conducted in the absence of any commercial or financial relationships that could be construed as a potential conflict of interest.
